# Metabolomic Evaluation of the Quality of Leaf Lettuce Grown in Practical Plant Factory to Capture Metabolite Signature

**DOI:** 10.3389/fpls.2018.00665

**Published:** 2018-06-27

**Authors:** Yoshio Tamura, Tetsuya Mori, Ryo Nakabayashi, Makoto Kobayashi, Kazuki Saito, Seiichi Okazaki, Ning Wang, Miyako Kusano

**Affiliations:** ^1^Graduate School of Life and Environmental Sciences, University of Tsukuba, Tsukuba, Japan; ^2^Central Research Institute for Feed and Livestock, National Federation of Agricultural Co-operative Associations, Tsukuba, Japan; ^3^RIKEN Center for Sustainable Resource Science, Yokohama, Japan; ^4^Graduate School of Pharmaceutical Sciences, Chiba University, Chiba, Japan; ^5^Keystone Technology, Yokohama, Japan; ^6^Graduate School of Environment and Information Sciences, Yokohama National University, Yokohama, Japan

**Keywords:** leaf lettuce, plant factory, hydroponic system, soil cultivation, metabolomics

## Abstract

Vegetables produce metabolites that affect their taste and nutritional value and compounds that contribute to human health. The quality of vegetables grown in plant factories under hydroponic cultivation, e.g., their sweetness and softness, can be improved by controlling growth factors including the temperature, humidity, light source, and fertilizer. However, soil is cheaper than hydroponic cultivation and the visual phenotype of vegetables grown under the two conditions is different. As it is not clear whether their metabolite composition is also different, we studied leaf lettuce raised under the hydroponic condition in practical plant factory and strictly controlled soil condition. We chose two representative cultivars, “black rose” (BR) and “red fire” (RF) because they are of high economic value. Metabolite profiling by comprehensive gas chromatography-mass spectrometry (GC-MS) and liquid chromatography-mass spectrometry (LC-MS) resulted in the annotation of 101 metabolites from 223 peaks detected by GC-MS; LC-MS yielded 95 peaks. The principal component analysis (PCA) scatter plot showed that the most distinct separation patterns on the first principal component (PC1) coincided with differences in the cultivation methods. There were no clear separations related to cultivar differences in the plot. PC1 loading revealed the discriminant metabolites for each cultivation method. The level of amino acids such as lysine, phenylalanine, tryptophan, and valine was significantly increased in hydroponically grown leaf lettuce, while soil-cultivation derived leaf lettuce samples contained significantly higher levels of fatty-acid derived alcohols (tetracosanol and hexacosanol) and lettuce-specific sesquiterpene lactones (lactucopicrin-15-oxalate and 15-deoxylactucin-8-sulfate). These findings suggest that the metabolite composition of leaf lettuce is primarily affected by its cultivation condition. As the discriminant metabolites reveal important factors that contribute to the nutritional value and taste characteristics of leaf lettuce, we performed comprehensive metabolite profiling to identify metabolite compositions, i.e., metabolite signature, that directly improve its quality and value.

## Introduction

For the stable supply of vegetables, plant factories use hydroponic cultivation that controls vegetable growth and development under closed environments by regulating important factors for plant growth such as the temperature, humidity, light, growing medium, and plant nutrition (Kozai, 2013). Many use artificial lighting for the cultivation of leafy vegetables and herbs. Because light-emitting diodes (LEDs) suppress heat, their energy requirement is lower than of other light sources such as fluorescent lamps. As plant factories involve higher initial financial investments and running costs than soil cultivation (Tokimasa and Nishiura, 2015), their cultivation conditions must be optimized to yield economically viable horticultural outputs.

Leaf lettuce (*Lactuca sativa* L. var crispa) is a popular leafy vegetable grown in plant factories. Optimization of hydroponic cultivation conditions has focused on increasing its yield (Touliatos et al., 2016) and on the accumulation of functional nutrients (Miyagi et al., 2017). Different light intensities influence the composition of important phytochemicals such as anthocyanins, carotenoids, chlorophylls, phenolics, and ascorbic acid in baby leaf lettuce (Li and Kubota, 2009; Samuoliene et al., 2013). Moreover, the soilless cultivation can be more efficient in term of nutrient requirements for vegetables growth than that of soil culture (Rouphael et al., 2004; Palermo et al., 2012). Although the metabolite composition of leaf lettuce grown under hydroponic cultivation has been investigated, a comparison of the primary and secondary metabolite profiles in lettuce grown under hydroponic- and soil cultivation conditions is still needed.

Metabolomics has been used to identify the effect of different cultivation environments on plants and for the quality evaluation of agricultural products (Kusano et al., 2011b; Zeng et al., 2014; Sung et al., 2015; Garcia et al., 2016; Ya-Qin et al., 2017). According to Lisec et al. (2006), plants can produce approximately 200,000 metabolites and specific metabolites are produced in different plant species (Kusano et al., 2011b, 2014a,b), even in cultivars and ecotypes (Sulpice et al., 2009). As mass spectrometry (MS)-based metabolomics yields highly sensitive results and facilitates high-throughput data acquisition, it has been combined with various types of analytical separation techniques, including gas chromatography (GC), liquid chromatography (LC), and capillary electrophoresis (CE) (Gowda and Djukovic, 2014). Since primary metabolites contribute to taste, integrated comprehensive GC-MS analysis and sensory evaluation were applied to determine the taste characteristics of green tea grown under various artificial light conditions, and bidimensional GC could provide extended metabolic phenotyping of natural variants in rice (Kusano et al., 2007; Miyauchi et al., 2017). LC-MS-based metabolite profiling aimed at detecting secondary metabolites including functional ingredients in foods and plants (Xie et al., 2008; Ma et al., 2013) revealed that environmental factors affected the accumulation of flavonoids in tea (Zhang et al., 2014). Comprehensive LC-MS analysis combined with ultra-high-performance LC (UHPLC) and quadrupole high-resolution time-of-flight MS (qTOF-MS) was used for secondary metabolite profiling to evaluate the quality of three artichoke cultivars and their commercial products (Farag et al., 2013). Furthermore, CE-MS-based metabolomics to analyze charged metabolites, i.e., amino acids and organic acids, has been utilized for quality control of agricultural products such as edamame beans, pork meat, and sake (rice spirit) (Sugimoto et al., 2010, 2012; Muroya et al., 2014). Additionally, the use of nuclear magnetic resonance (NMR) spectroscopy in plant metabolomics study also complements analytical technique choices in providing robust and reproducible metabolic data (Sobolev et al., 2005; Pereira et al., 2014; Sekiyama et al., 2017).

Metabolite profiling is a promising analytical method for the elucidation of taste characteristics and for the functional evaluation of agricultural products because unlike targeted analysis, it returns a variety of component profiles for many samples. Moreover, multi-MS-based metabolite profiling yields wider coverage for the detection of a large variety of metabolites than single chromatographic techniques combined with MS (Arbona et al., 2009; Lee et al., 2014; Sung et al., 2015). It not only detects variations in taste-related metabolites elicited by differences in cultivation conditions, but also contributes to the identification of quantitative changes in specialized metabolites in vegetables. Taken together, collection of metabolite alterations data of leaf lettuce from different cultivation systems is very important for clarifying their quality.

**FIGURE 1:**
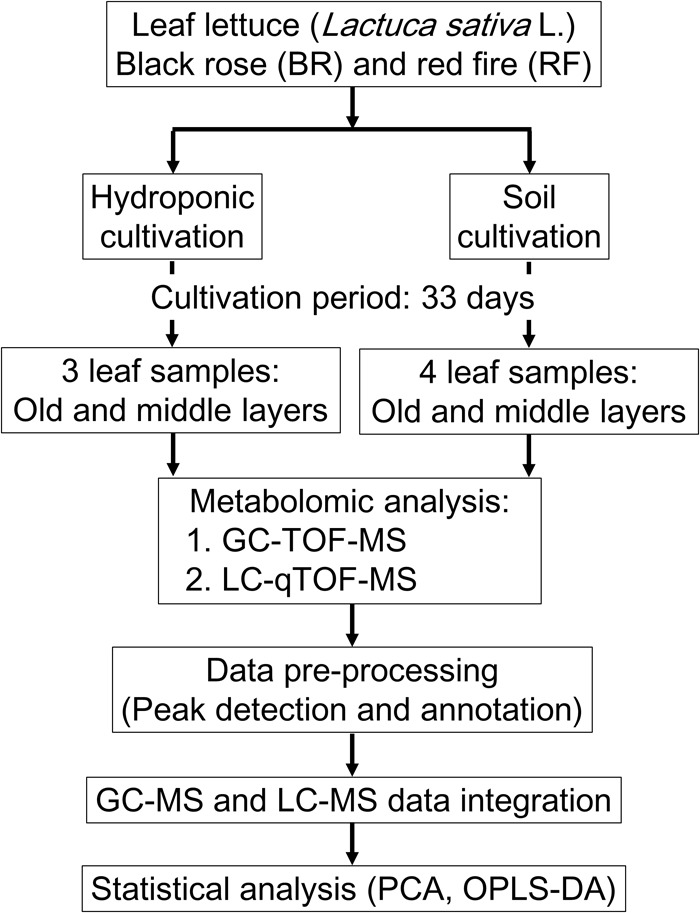
Workflow for the metabolomic evaluation of leaf lettuce grown under hydroponic- and soil cultivation methods. The leaf samples from hydroponic and soil cultivations were 3 and 4 biological replicates, respectively.

We performed GC-MS- and LC-MS analysis for the comprehensive capture of cultivation-specific- and taste-related metabolites in leaf lettuce (**Figure 1**). To differentiate the metabolite composition, including primary and secondary metabolites, of two leaf lettuce cultivars, black rose (BR) and red fire (RF), grown under hydroponic or fertilized soil conditions, including light sources and nutrient, we performed integrated MS-based metabolite profiling to capture metabolite signature for BR and RF, and thus to establish their quality indexes.

## Materials and Methods

### Plant Materials and Growth Condition

Two cultivars of *L. sativa* L. cv. BR and RF were grown for 33 days under hydroponic or soil conditions. The soil was treated with optimized concentrations of liquid fertilizer. The BR and RF seeds were purchased from Kaneko Seed Co. (Japan) and Takii Seed Co., Ltd. (Japan), respectively.

For hydroponic cultivation of BR and RF lettuce we applied the methods used by Keystone Technology Inc. (Japan). The conditions were: temperature, 22°C; 16 h light/8 h dark cycle (16 l/8 days); photosynthetic photon flux density (PPFD), 150 μmol m^-2^s^-1^ from light-emitting diode (LED) light (peak wavelength: blue = 460 nm, green = 525 nm, red = 660 nm). For soil cultivation the growth-chamber (LPH-350S, Nippon Medical and Chemical Instruments Co., Ltd., Japan) conditions were: temperature, 24°C; 16 l/8 days; PPFD, 140 μmol m^-2^ s^-1^ from white LED light, 2–3 times per week watering (Tamoi et al., 2017). The leaves of each plant were counted starting with the smallest leaf and the leaf growth stages were recorded as “young,” “middle” and “old” (Kusano et al., 2011a). The collected leaf samples were as follows: hydroponic cultivation; 2 cultivars × 2 sampling positions × 3 biological replicates, soil cultivation; 2 cultivars × 2 sampling positions × 4 biological replicates. In total, there were 28 leaf samples (**Figure 1**). Leaf samples were collected in bulk from 4 sites on leaves in the middle- and old stage using an 8-mm leaf punch disk (Fujiwara Scientific Company Co., Ltd., Japan) and immediately frozen in liquid nitrogen. Samples were stored at -80°C until analysis.

### Metabolite Profiling by GC-TOF-MS

For the extraction of 25-mg leaf samples (fresh weight) we used 1 ml methanol/chloroform/H_2_O (3:1:1, v/v/v) containing 10 stable isotope references. The extraction was performed for 10 min at 15 Hz and 4°C using a mixer mill (Retsch MM301, Retsch GmbH, Germany) (Kusano et al., 2007). The mixture was then centrifuged, the supernatant was placed in a glass insert vial, and evaporated to dryness in a vacuum concentrator (Savant SPD2010 SpeedVac, Thermo Fisher Scientific Inc., United States). The dry extracts were derivatized using 30 μl of methoxylamine hydrochloride-HCl (20 mg/ml in pyridine) for 23 h at room temperature, then for 1 h in 30 μl of N-methyl-N-trimethylsilyltrifluoroacetamide (MSTFA) at 37°C with shaking; heptane (30 μl) was added to the extract mixture.

The extract (1 μl) was injected in into an Agilent 6890N GC instrument (Agilent Technologies, United States) via a CTC CombiPAL autosampler (CTC Analytics, Switzerland) for primary metabolite profiling. For separation we used an Rxi-5 Sil MS column [RESTEK, United States, inner diameter (ID), 30 m × 0.25 mm; film thickness, 0.25 μm]. Helium was the carrier gas delivered at a constant flow rate of 1 ml/min. The initial GC oven temperature was set at 80°C for 2 min, raised to 320°C at a rate of 30°C/min, and then held constant for 3.5 min. Data acquisition was performed on a Pegasus IV TOF MS instrument (LECO Corp., United States); the acquisition rate and range were 30 spectra/s and *m/z* 60–800, respectively. Alkane standard mixtures (C8–C20 and C21–C40, Sigma-Aldrich, Japan) were used for calculating the retention index (RI).

### Metabolite Profiling by LC-TOF-MS

The dried samples were extracted (7 min, 18 Hz, 4°C) with 150 μl of 80% MeOH and zirconia beads containing 2.5 μM lidocaine and 10-Camphorsulfonic acid per mg dried weight using a mixer mill. Zirconia beads were used to increase extraction efficiency in particularly for cationic compounds, e.g., anthocyanins. After centrifugation, the supernatant was filtered using an Oasis HLB μElution plate (Waters Co., United States) (Nakabayashi et al., 2014). Extract (1 μl) was injected in into a Waters Acquity UPLC instrument coupled with a Waters Xevo G2 QTOF-MS instrument for metabolite profiling. The analytical conditions were LC column: Acquity-bridged ethyl hybrid (BEH) C18 (ID, 100 mm × 2.1 mm; 1.7 μm particle diameter; Waters); solvent system: solvent A (water including 0.1% formic acid); solvent B (acetonitrile including 0.1% formic acid); gradient program: 99.5% A/0.5% B, 0 min; 99.5% A/0.5% B, 0.1 min; 20% A/80% B, 10 min; 0.5% A/99.5% B, 10.1 min; 0.5% A/99.5% B, 12.0 min; 99.5% A/0.5% B, 12.1 min; 99.5% A/0.5% B, 15.0 min; flow rate: 0.3 ml/min at 0 min, 0.3 ml/min at 10 min, 0.4 ml/min at 10.1 min, 0.4 ml/min at 14.4 min, and 0.3 ml/min at 14.5 min; column temperature: 40°C; MS detection: capillary voltage, +3.0 keV; cone voltage, 25.0 V; source temperature, 120°C; desolvation temperature, 450°C; cone gas flow, 50 l/h; desolvation gas flow, 800 l/h; collision energy: 6 V; mass range: *m/z* 50–1500; scan duration: 0.1 s; interscan delay: 0.014 s; data acquisition: centroid mode; polarity: positive/negative; Lockspray (leucine enkephalin); scan duration: 1.0 s; interscan delay: 0.1 s. MS/MS data were acquired in ramp mode under the following analytical conditions: (1) MS: mass range, *m/z* 50–1500; scan duration, 0.1 s; inter-scan delay, 0.014 s; data acquisition, centroid mode; polarity, positive/negative; and (2) MS/MS: mass range, *m/z* 50–1500; scan duration, 0.02 s; inter-scan delay, 0.014 s; data acquisition, centroid mode; polarity, positive/negative; collision energy, ramped from 10–50 V. In this mode, MS/MS spectra of the top 10 ions (>1000 counts) in an MS scan were automatically obtained. If the ion intensity was below 1000, we did not perform MS/MS data acquisition but moved on to the next top 10 ions. Data acquisition was with Progenesis CoMet (Nonlinear Dynamics). Peak normalization was with lidocaine (positive mode) and 10-camphorsulfonic acid (negative mode).

### Data Pre-processing

Non-processed data (NetCDF format) from GC-TOF-MS were exported to MATLAB (Mathworks, United States); custom scripts were used for data normalization, baseline correction, and subsequent analysis. Lastly, processed data obtained from hyphenated data analysis (HDA) were identified or annotated using an in-house metabolite library in PRIMe (Platform for RIKEN Metabolomics^1^), and the library in the Golm Metabolome Database (GMD) (Jonsson et al., 2005, 2006). Peaks were normalized with the cross-contribution compensating multiple standard normalization (CCMN) method (Redestig et al., 2009). Chemical assignment of data acquired from LC-TOF-MS was performed using reported MS- or MS/MS data (Wu and Prior, 2005; Abu-Reidah et al., 2013) and the KNApSAcK database (keyword: *Lactuca sativa*). The *m/z* values were set as monoisotopic masses [(M+H)^+^ or (M-H)^-^] under a tolerance match limit of 0.01 Da.

### Statistical Analysis

To determine the effect of the different cultivation conditions on the metabolite profile of leaf lettuce we performed multivariate statistical analysis including principal component analysis (PCA) and orthogonal partial least square-discriminant analysis (OPLS-DA) of the unit variance scaled and log-_10_-transformed data matrix obtained from GC- and UPLC**-**TOF-MS using SIMCA-P 14 (Umetrics, Sweden). Significant metabolites in leaf lettuce were evaluated based on their differing variable importance in projection (VIP) values calculated with OPLS-DA and *Q*-values adjusted via the false discovery rate (FDR) approach (Benjamini and Hochberg, 1995) using R package samr^2^. The effect of the different cultivation methods on the resulting common metabolites in the cultivars was visualized on the Venn diagram (VENNY ver.2.1^3^).

## Results

### Experimental Design

**FIGURE 2:**
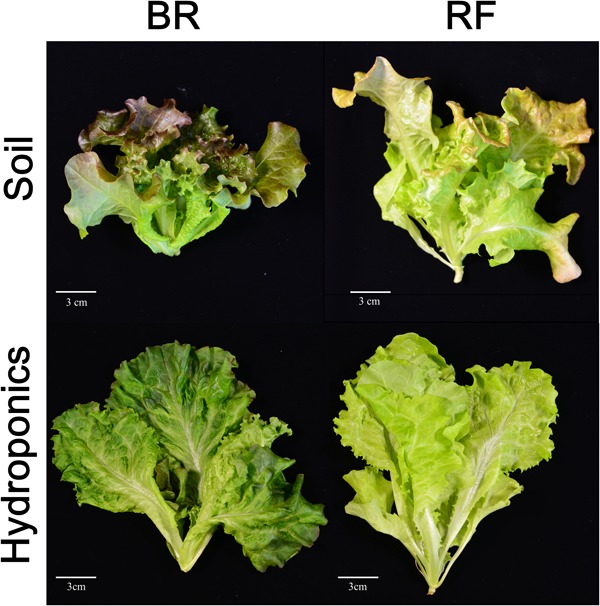
Black rose (BR) and red fire (RF) lettuce leaves grown under the two cultivation conditions. White bar, 3 cm.

To investigate the effect of different cultivation conditions, cultivars, and leaf positions on the metabolite composition of leaf lettuce, we studied BR and RF cultivars raised for 33 days under the strictly controlled soil and hydroponic conditions (**Table 1** and **Figure 1**). As metabolite profiles are largely affected the extent of visual phenotypes (Fiehn et al., 2000), we optimized soil condition for minimizing differences of visual phenotypes when compared to lettuce phenotypes harvested from the practical plat factory. The same type of liquid fertilizer was applied under both conditions and the light intensity was almost the same. Different concentration of the fertilizer was used for each cultivation because appearance and growth was different when we used the similar concentration for both cultivations (data not shown). Comparison of their phenotypes showed that the BR and RF lettuce leaves exposed to soil and hydroponic conditions were distinct (**Figure 2**). Soil-based cultivation yielded smaller colored leaves than hydroponic farming. Soil-grown BR and RF leaves were of the typical green color with dark- (BR) and bright-red pigments (RF) at the tip. For GC- and LC-MS, tissue samples from leaves in the old- and middle growth stage were collected from the same plant using an 8-mm leaf disk. The metabolites obtained by GC- and LC-MS were identified or annotated, and the metabolite profile data were then integrated for data interpretation by multivariate statistical analysis.

**Table 1:** Soil- and hydroponic-cultivation conditions.

Parameter	Soil	Hydroponics
LED light source wavelength (nm)	White (400–800)	Blue (460), green (525), red (660)
Light intensity (PPFD)^∗^	140	150 (blue, 23%; green, 5%; red, 72%)
Liquid fertilizer (ppm)	NH_3_-N, 0.50; NO_3_-N, 25.00; P, 9.00; K, 56.00; Mg, 11.00; Mn, 0.05; B, 0.20; trace (Cu, Zn, Mo, Fe, Ca)	NH_3_-N, 1.67; NO_3_-N, 83.33; P, 30.00; K, 186.67; Mg, 36.67; Mn, 0.17; B, 0.67; trace (Cu, Zn, Mo, Fe, Ca)

*^∗^PPFD, photosynthetic photon flux density (μmol m^-*2*^s^-*1*^).*

### Metabolite Profiling of Leaf Lettuce Grown Under Different Cultivation Conditions

To investigate metabolite changes, including primary and secondary metabolites, in BR and RF lettuce grown under different cultivation conditions, we harvested 28 leaf samples from each cultivar for comprehensive GC- and LC-MS analyses. GC-MS detected 223 peaks; 101 were identified- or provisionally identified primary metabolites. Positive- and negative-ionization mode-LC-MS followed by an MS/MS library search made it possible to annotate 2 of 30- and 30 of 65-detected compounds, respectively (Supplementary Table 1). Unsupervised multivariate statistical analysis by PCA was then performed to visualize the extent of metabolite changes elicited in the cultivars, under the different cultivation conditions, and in the leaf position.

**FIGURE 3:**
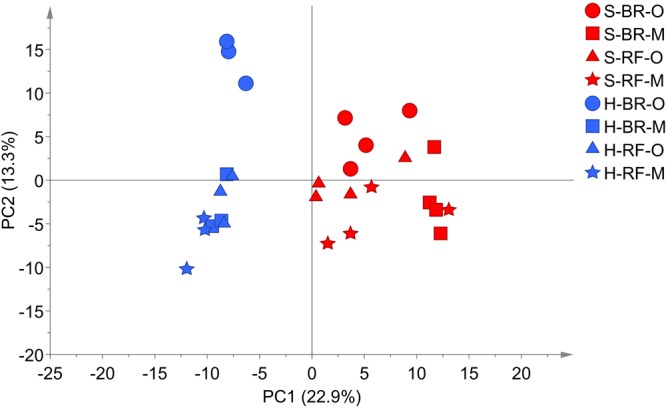
PCA score scatter plot of metabolites derived from black rose (BR) and red fire (RF) lettuce leaves (O, old layer; M, middle layer) grown under hydroponic (H) and soil (S) cultivation conditions. The leaf samples from hydroponic and soil cultivations were 3 and 4 biological replicates, respectively. The normalized data matrix (318 peak areas × 28 leaf samples) obtained from GC and LC-MS was used.

**FIGURE 4:**
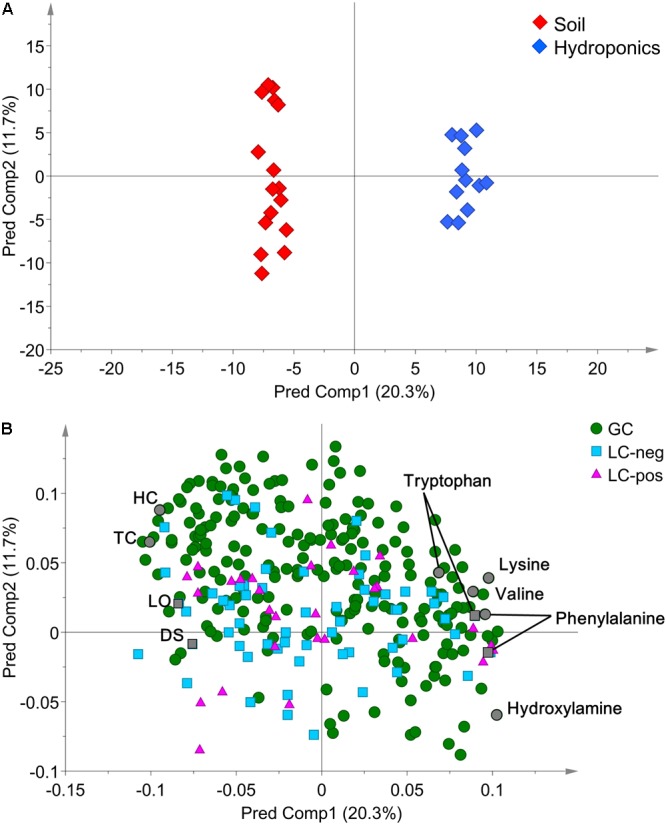
**(A)** OPLS-DA score scatter- and **(B)** loading plots of metabolites derived from 12 hydroponically- and 16 soil-grown lettuce leaves (number of samples: hydroponic cultivation; 2 cultivars × 2 sampling positions × 3 biological replicates, soil cultivation; 2 cultivars × 2 sampling positions × 4 biological replicates). The normalized data matrix (318 peak areas × 28 leaf samples) obtained by GC and LS-MS was used. HC, 1-hexacosanol; TC, 1-tetracosanol; LO, lactucopicrin-15-oxalate; DS, 15-deoxylactucin-8-sulfate.

In detail, “cultivation conditions” could differentiate on the first principal component, PC1 (22.9%), in which soil-based cultivation was recorded in the positive quadrant and hydroponic in the negative (**Figure 3**). Except for samples obtained from old-layer BR leaves grown hydroponically (H-BR), the plots of the other leaves were not clearly separated. This observation suggests that there is little difference in the metabolite composition of the cultivars and of leaves sampled at different growth stages. We next identified the discriminant metabolites in leaf lettuce by OPLS-DA. The score scatter plot in **Figure 4A** shows that the samples were clearly separated by the cultivation condition [PredComp1 = 20.5%, overall predictive performance of the model = R2 (Y): 0.99%, Q2 (cum) = 0.92%]. Therefore, the cultivation conditions had more impact on metabolite changes than the cultivar-type or the leaf growth stage.

### Discriminant Metabolites in Soil- and Hydroponic-Cultivated Leaf Lettuce

The discriminant metabolites for our quality index of leaf lettuce were screened from all 318 metabolites based on the selection criteria of VIP>1.0 and FDR<0.05 (**Figure 4B** and Supplementary Table 2). We then compared changes in the metabolite profiles of hydroponically- and soil-grown lettuce. Leaves harvested after soil cultivation contained more typical lettuce sesquiterpene lactones and fatty acid-derived alcohols [log_2_-fold change (FC) ranging from -3.37 to -1.45] than hydroponically grown leaf lettuce which contained more amino acids (log_2_-FC ranging from 0.84 to 4.25).

**FIGURE 5:**
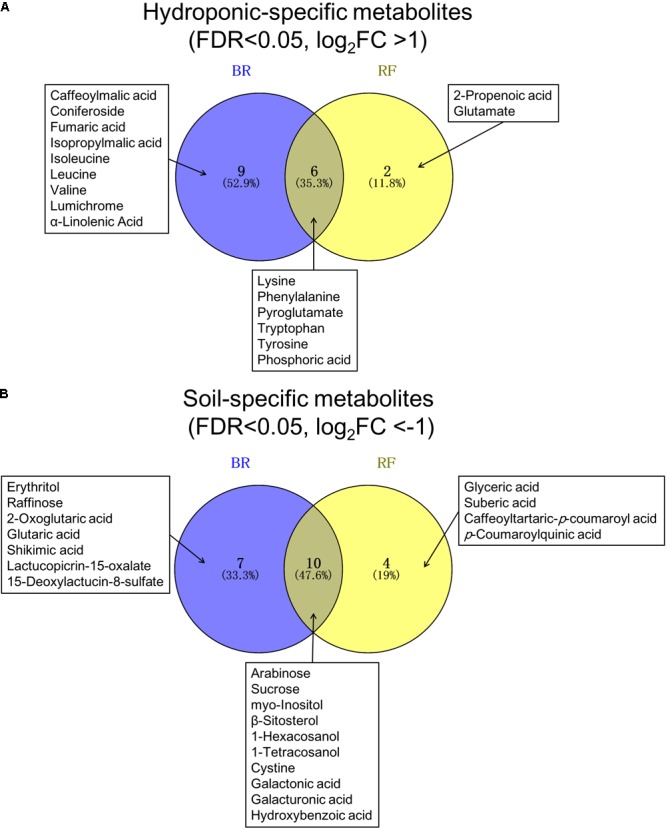
Venn diagram of **(A)** hydroponic-specific- and **(B)** soil-specific metabolites in black rose (BR) and red fire (RF) lettuce leaves. The false discovery rate (FDR) and log_2_-fold changes (FC) were calculated with soil cultivation as the control condition. Significant metabolites of the two lettuce cultivars were identified using a threshold of FDR<0.05 and | log2-FC| > 1.

**FIGURE 6:**
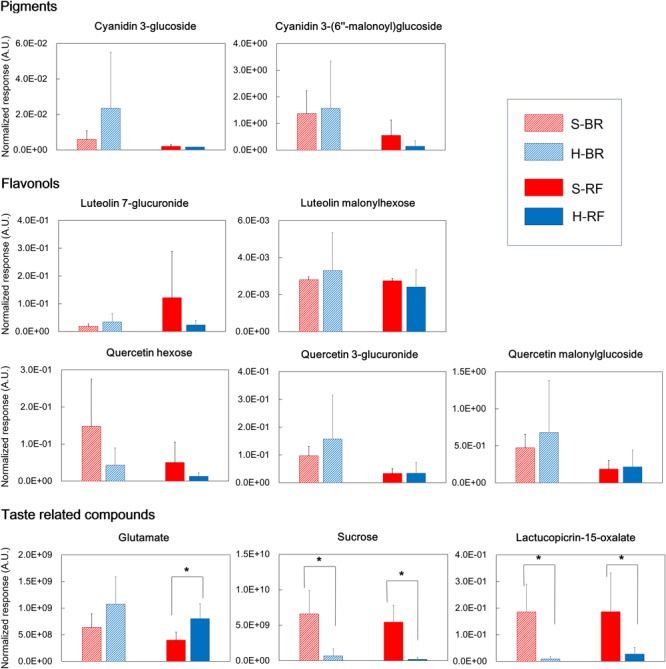
Quality index metabolites, represented by pigments, flavonols, and taste-related compounds, of Black rose (BR) and red fire (RF) lettuce leaves grown under hydroponic (H) and soil (S) cultivation conditions (number of samples: 6 × H-BR, 6 × H-RF, 8 × S-BR and 8 × S-RF). (^∗^indicates significant difference at *p* < 0.05 analyzed by Student’s *t*-test).

In both cultivars we looked for metabolites that differed when they were grown under the two different conditions. We extracted discriminant metabolites that showed FDR<0.05 and |log_2_-FC| >1; our findings are presented in a Venn diagram (**Figure 5** and Supplementary Tables 3, 4). When GC-MS and LC-MS detected the same metabolite, we selected the metabolite peak with the higher VIP value. The Venn diagram of metabolites that accumulated under hydroponic conditions (log_2_-FC>1) shows that common metabolites in BR and RF were amino acids (lysine, phenylalanine, pyroglutamate, tryptophan, and tyrosine) and phosphoric acid (**Figure 5A**). Soil-specific metabolites (log_2_-FC<-1) were sugars, organic acids, sesquiterpenes, and fatty acid-derived alcohols. Common metabolites in soil-grown BR and RF were arabinose, sucrose, *myo*-inositol, *β*-sitosterol, 1-hexacosanol, 1-tetracosanol, cystine, galactonic acid, galacturonic acid, and hydroxybenzoic acid (**Figure 5B**). The cultivar-specific metabolites in BR leaves grown hydroponically were amino acids (isoleucine, leucine and valine), caffeoylmalic acid, and coniferoside; in RF leaves grown hydroponically they were 2-propenoic acid and glutamate (**Figure 5A**). BR-specific metabolites obtained from plants grown in soil were sugars (erythritol and raffinose), organic acids (2-oxoglutaric, glutaric, and shikimic acids), and sesquiterpenes (lactucopicrin-15-oxalate and 15-deoxylactucin-8-sulfate); RF-specific metabolites included organic acids (glyceric and suberic acids) and phenolics (caffeoyltartaric-*p*-coumaroyl and *p*-coumaroylquinic acids) (**Figure 5B**). The different cultivation systems did not significantly alter the content of pigment and flavonol compounds in leaf lettuce, while these compounds tended to be accumulated in BR leaves (**Figure 6**). The application of the two cultivation methods could significantly differentiate (*p* < 0.05) taste-related compounds that might influence the sensory acceptance of lettuce, including glutamate (umami) (Halpern, 2000; Hounsome et al., 2008; Kurihara, 2009), sucrose (sweetness), and lactucopicrin-15-oxalate (bitterness) (**Figure 6**). Thoroughly, RF leaves from hydroponic cultivation had significantly more glutamate content than that of soil-cultivated RF leaves. Then again, both BR and RF leaves grown hydroponically had significantly less sucrose and lactucopicrin-15-oxalate levels than leaves from soil cultivation.

## Discussion

### Effect of Cultivation Conditions on Leaf Lettuce Growth

The leaves of BR and RF lettuce grown for 33 days in soil or under hydroponic conditions exhibited different morphological characteristics, i.e., their color and shape, but similar size (**Figure 2**). It has been reported that metabolite profiles largely affect from differences that is considered to be derived from difference of growth stages as well as visible phenotypes (Kusano et al., 2011a; Clevenger et al., 2015; Li et al., 2016). Soil-cultivated leaves had more reddish appearance than leaves grown hydroponically. Under both growth conditions the BR leaves were reddish and more darkly green than RF leaves, suggesting leaf pigmentation is affected by cultivar characteristics (Caldwell and Britz, 2006). According to Stutte et al. (2009) and Son and Oh (2013), the combination of red and blue LED light yields red leaf lettuce while the sole exposure to red LED light failed to yield pigmented leaves. These differences in appearance, including the leaf morphology and color, may influence sensorial preferences and the acceptance RF and BR lettuce (McWatters et al., 2002). As plant pigmentation reflects the level of chlorophyll and other secondary metabolites such as carotenoid and anthocyanin, we hypothesized that environmental- and cultivar-related factors impact the primary and secondary metabolite composition associated with metabolic pathways (Sass-Kiss et al., 2005; Caldwell and Britz, 2006). Therefore, we used comprehensive GC- and LC-MS-based metabolic profiling to identify specific metabolites related with the BR and RF lettuce phenotype except for chlorophylls and carotenoids. Because the MS-based metabolomics platform cannot detect these compounds.

### Metabolomic Evaluation of the Quality of Leaf Lettuce Grown Under Different Cultivation Conditions

We demonstrate that the cultivation condition exerts greater influence on the metabolite composition of leaf lettuce than the cultivar type or the leaf position. Environmental factors of cultivation systems can contribute to the elicitation of taste- and nutrition-related compounds in various plants (Ohashi-Kaneko et al., 2007; Zhang et al., 2014). Okazaki et al. (2008), who examined the influence of the primary metabolite composition and of nitrogen availability in two spinach cultivars grown under different inorganic nitrogen concentrations, found that the efficiency of their nitrogen use was not the same.

We varied the light source intensity and the liquid fertilizer concentration in our study of RF and BR lettuce grown hydroponically and in soil. Miyagi et al. (2017) reported that red LED radiation combined with high concentrations of carbon dioxide and liquid fertilizer increased the biomass and the level of some amino acids in head lettuce grown hydroponically. Nitrogen, the main component of liquid fertilizer, impacts the plant metabolism. We found higher concentrations of amino acids (leucine, isoleucine, tryptophan, tyrosine, and phenylalanine) in the leaves of hydroponically- than soil-grown BR and RF lettuce. This phenomenon might also be affected by the applied nutritional values in both cultivation systems wherein more nitrogen nutrition was provided for hydroponic condition than in soil cultivation (**Table 1**). Due to the elicitation of higher amino acid levels, hydroponic cultivation may result in stronger “umami” perception than soil cultivation (Morris et al., 2007; Wang et al., 2016). On the other hand, the accumulation of sugars, fatty acid-derived alcohols, and β-sitosterol was lower in hydroponically- than soil-grown lettuce leaves. Our findings agree with earlier reports that the accumulation of sugars may be affected by environmental factors or stress such as light- and nutrient-conditions (Okazaki et al., 2008; Rosa et al., 2009; Miyagi et al., 2017). The level of leaf epidermal wax compounds such fatty acid-derived alcohols and β-sitosterol might be decreased in lettuce leaves grown in a hydroponic culture system (Baker et al., 1979). Also, the three-times shorter shelf life of hydroponically grown leaf lettuce may be attributable to lower dehydration (Manzocco et al., 2011), suggesting that the decreased level of fatty acids and sterols we observed in hydroponically grown lettuce contributed to the change in its leaf epidermal composition. Additionally, increased levels of fatty acids were considered to reflect stress of lettuce in soil (Yang and Ohlrogge, 2009).

To evaluate the effect of environmental growth conditions on RF and BR lettuces, we compared their metabolite profiles and extract metabolite signature for developing a quality index. Compared with hydroponically grown lettuce, the accumulation of sesquiterpene lactones and phenolics was markedly greater in BR- and RF-lettuce leaves harvested after soil cultivation. Seo et al. (2009) reported that the correlation between mineral nutrients intake and producing of sesquiterpene lactones in leaf lettuce. Exposure to short-wavelength light (blue LED and UV**-**B treatments) elicits an increase in phenolics, flavonoids, and the pigment content in lettuce leaves (Ouzounis et al., 2015) and in *Arabidopsis thaliana* (Kusano et al., 2011c) and our light conditions may have exerted a similar effect. Caldwell and Britz (2006) suggested that differences in the photosensitivity trait among cultivars changes the metabolite composition in leafy vegetables. The higher level of phenolics in soil- than hydroponically grown RF lettuce may increase its antioxidant and antimutagenic effects but render it more bitter-tasting and more astringent (Ren et al., 2001; Altunkaya et al., 2016; Filippo D’Antuono et al., 2016). BR-specific metabolites such as sesquiterpene lactones found in soil-grown plants may also enhance its bitter taste (Chadwick et al., 2016; Filippo D’Antuono et al., 2016). On the other hand, the sugar components in these soil-grown lettuces may mask their bitter taste and enrich their taste complexity (Beck et al., 2014; Chadwick et al., 2016).

## Conclusion

Our study shows that the two cultivation methods greatly affect the metabolite profile of RF and BR leaf lettuce, including the metabolites responsible for their taste and their functional ingredients (e.g., amino acids and phenolic compounds). These metabolites may contribute to the overall quality and sensorial perception of leaf lettuce grown under hydroponic and soil cultivation methods (Manzocco et al., 2011; Mampholo et al., 2016). Metabolomic elucidations for lettuce have been conducted using various analytical techniques, including GC-MS, LC-MS, CE-MS, and NMR analyses (Pereira et al., 2014; Garcia et al., 2016; Zhao et al., 2016; Hurtado et al., 2017; Miyagi et al., 2017; Kitazaki et al., 2018). These techniques enabled to detect most of nutritional and pigment metabolites in the evaluated lettuce. Thus, metabolomic data can be used as an important basis for further detailed assessment of cultivation systems and horticulture products not only for lettuce, but for other vegetables. As the next step, it will be needed to evaluate which growth factor(s) are essential to affect phytochemical accumulation as well as yield in plant factories. We document that integrated metabolic profiling is a powerful tool for the comprehensively evaluation of the quality of leaf lettuce. Studies are underway to examine the effect of the light properties and of the liquid fertilizer concentration on the quality and nutritional value of leaf lettuce.

## Author Contributions

MK initiated the study conception. MK, KS, and SO designed the analysis. YT performed the statistical analysis and drafted the manuscript. TM, RN, and MKo worked on data acquisition and analysis. MK supervised the study. SO and NW created and provided the samples. YT and MK wrote the manuscript and interpreted biological meaning of the results.

## Conflict of Interest Statement

The authors declare that the research was conducted in the absence of any commercial or financial relationships that could be construed as a potential conflict of interest.

## Footnotes

1 http://prime.psc.riken.jp

2 https://cran.r-project.org/web/packages/samr/index.html

3 http://bioinfogp.cnb.csic.es/tools/venny/
