# Supplementary material for: Metabolomic Evaluation of the Quality of Leaf Lettuce Grown in Practical Plant Factory to Capture Metabolite Signature
Source: Front Plant Sci. 2018 Jun 27;9:665. doi: 10.3389/fpls.2018.00665 (PMC6030546; doi:10.3389/fpls.2018.00665)
Supplement: Supplementary file 5 [file Data_Sheet_1.DOCX]

Supplementary Material

Metabolomic evaluation of the quality of leaf lettuce grown under strictly-controlled conditions and different cultivation methods

Yoshio Tamura, Tetsuya Mori, Ryo Nakabayashi, Makoto Kobayashi, Kazuki Saito,

Seiichi Okazaki, Wang Ning, Miyako Kusano,*

*** Correspondence:** Miyako Kusano: kusano.miyako.fp@u.tsukuba.ac.jp

# Supplementary Tables

**Supplementary Table 1.**

Dataset of metabolites obtained from GC- and LC-MS analyses.

**Supplementary Table 2.**

Discriminant metabolites in RF and BR leaves grown under hydroponic- and soil-cultivation.

**Supplementary Table 3.**

Specific metabolites in black rose (BR) lettuce leaves grown under hydroponic- and soil-cultivation.

**Supplementary Table 4.**

Specific metabolites in red fire (RF) lettuce leaves grown under hydroponic- and soil-cultivation.
